# AFP and CA-125 as an accurate risk factor to predict eye metastasis in hypertension patients with liver carcinoma: A STROBE-compliant article

**DOI:** 10.3389/fgene.2022.1010903

**Published:** 2022-09-19

**Authors:** Jing Tang, Li-Juan Zhang, Min Kang, Rong Huang, Hui-Ye Shu, Hong Wei, Jie Zou, Yi-Cong Pan, Qian Ling, Yi Shao

**Affiliations:** ^1^ Department of Oncology, The First Affiliated Hospital of Nanchang University, Jiangxi Center of National Ocular Disease Clinical Research Center, Nanchang, Jiangxi, China; ^2^ Department of Ophthalmology, The First Affiliated Hospital of Nanchang University, Jiangxi Center of National Ocular Disease Clinical Research Center, Nanchang, Jiangxi, China

**Keywords:** AFP, CA-125, ocular metastasis, primary liver cancer, hypertension, history of hepatitis B

## Abstract

**Purpose:** In this study, we analyzed the differences between hypertension patients with ocular metastasis of liver cancer and those with metastases to other sites, the correlation between history of HBV and liver cancer metastasis, and independent risk factors for ocular metastasis.

**Methods:** We used treatment records from 488 patients with metastases of primary liver cancer from August 2001 to May 2015, divided into two groups based on metastatic sites: OM (ocular metastasis) and NOM (non-ocular, other sites of metastasis) groups. The Student’s *t-*test and Chi-square test were used to assess the significance of differences between the groups and define the relationship between history of HBV and ocular metastasis of liver cancer. Binary logistic regression analysis was used to identify indicators of ocular metastasis of liver cancer and receiver operating curve (ROC) analyses to estimate their diagnostic value.

**Results:** No significant differences in sex, age, tumor stage, pathological type, or treatment were identified between the OM and NOM groups, while the prevalence of HBV was higher in the former than that in latter. Binary logistic regression demonstrated that AFP and CA-125 were independent indicators of liver metastasis (both *p* < 0.001). ROC curve analyses generated cut-off values for AFP and CA-125 of 957.2 ng/ml and 114.25 U/ml, respectively, with corresponding AUC values of 0.739 and 0.810. The specificity of the combination of AFP and CA-125 was higher than either factor separately.

**Discussion:** To explore the diagnostic value of AFP and CA125 in predicting the development of ocular metastases of hypertensive patients with liver cancer, which will help us to diagnose the occurrence and development of the disease more accurately and make the best clinical diagnosis and treatment measures.

## Introduction

Hypertension is a chronic disease with functional or organic damage of heart, brain, kidney, and other organs, which brings a huge health burden to the society. Primary liver cancer (PLC) is a common disease worldwide, particularly in developing countries (Zhang et al., 2015). Hepatocellular carcinoma (HCC) is the main pathological type of PLC, and exhibits high malignancy and strong invasiveness, resulting in poor prognosis and a heavy economic burden of treatment (Wang et al., 2016). Worldwide, patients with hepatitis B virus (HBV) infection exceed 300 million, and many liver diseases, including liver cancer, can be secondary to HBV infection (Liang, 2009). Moreover, chronic hepatitis virus infection not only affects the liver, but also extrahepatic organs, leading to severe extrahepatic lesions such as dry eye, Mooren’s ulcer, and retinopathy. HBV infection is clearly linked to dry eye syndrome (Tsoumani et al., 2013). Extrahepatic metastasis is an indicator of prognosis in patients with PLC, and different metastatic sites are associated with distinct survival rates (Wu et al., 2017). A survey of 419 patients with HCC who had extrahepatic metastases found that the most common sites of extrahepatic metastases are lung, bone, lymph node, and adrenal gland in that order (Aino et al., 2014). The eye is a rare site of distant hepatic metastasis of PLC, and most patients with ocular metastases also have metastases at other sites. Hypertensive patients are prone to arteriosclerosis, which leads to liver blood supply insufficiency and liver function damage. Related clinical symptoms can cover up the signs of liver cancer, and the early clinical symptoms of HCC are not obvious; hence, patients can be completely unaware of their disease progression and even metastasis (McMahon et al., 2016). Once a distant metastasis occurs, treatment is extremely difficult, and patient prognosis is poor. At present, the diagnosis of PLC mainly depends on imaging [ultrasound and magnetic resonance imaging (MRI)] (Cassinotto et al., 2017), clinicopathological correlation, and application of immunohistochemical markers (Vyas and Jain, 2018); however, these methods are inefficient for early detection of metastases, hence a simple and effective clinical diagnostic indicator is needed to improve the early diagnosis rate of ocular metastasis from PLC.

## Materials and methods

### Study design

**FIGURE 1:**
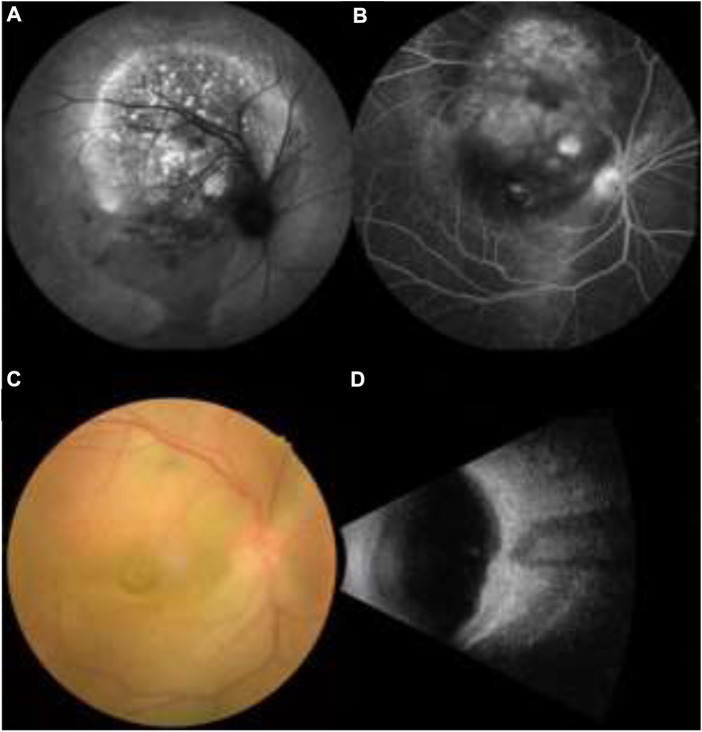
**(A**,**B)** Fundus fluorescein angiography in patients with primary liver cancer, **(C**,**D)** Funduscopy in patients with primary liver cancer.

This study met the requirements of the Declaration of Helsinki and was licensed by the Medical Ethics Committee of the First Affiliated Hospital of Nanchang University. The study design followed relevant regulations and guidelines. Hypertensive patients with PLC metastases (*n* = 488), admitted from August 2001 to May 2015, were enrolled in the study. The inclusion criteria are: 1) hypertension (systolic blood pressure >140 mmHg or diastolic blood pressure >90 mmHg); 2) without contraindications in MRI, CT and other imaging examinations; 3) Canceration of liver tissue; 4) ocular metastasis of hepatocellular carcinoma (Figure 1). Diagnosis was determined by imaging [ultrasound, computed tomography (CT), MRI] and histopathological biopsy. A review of the medical records of the patients revealed that those with ocular metastases of liver cancer also had metastases at other sites. Therefore, patients were divided into two groups, according to the tumor metastasis sites: OM (PLC with ocular metastasis) and NOM group (PLC with non-ocular, but other metastasis sites) groups. Exclusion criteria for the OM group were: 1) primary malignant tumor of the eye; 2) benign tumor of the eye; 3) primary liver cancer patients with metastases in other sites (intrahepatic, portal system, lung, bone, etc.).

### Data collection

Relevant clinical data were collected retrospectively from the medical records of each subject, including sex, age, history of HBV, clinical stage, pathological type, treatment methods, and other clinical features, serum markers (AFP, CEA, CA-125, CA-199, CA-153, and CA-724), blood lipid indicators {lipoprotein a [Lp(a)], apolipoprotein B (ApoB), apolipoprotein A1 (ApoA1), low-density lipoprotein (LDL), high-density lipoprotein (HDL), triglycerides (TG), and total cholesterol (TC)}, serum calcium concentration, hemoglobin concentration, ferritin, alkaline phosphatase (ALP), and other serum indicators. All clinical data were collected at initial diagnosis.

### Statistical analysis

Student’s *t-*tests and Chi-square tests were used to evaluate the significance of differences in clinical characteristics between the OM and NOM groups. Binary logistic regression analysis was used to identify indicators of ocular metastasis of liver cancer. ROC curve analysis was conducted and the area under the curve (AUC) used to assess the accuracy of risk factors as diagnostic indicators. *p* < 0.05 was considered statistically significant. SPSS 25.0 (SPSS, IBM, United States), MedCalc 19.0.5 (MedCalc Ostend, Belgium), and Excel 2019 (Microsoft Corp, Redmond, WA, United States) software were used for statistical analyses.

## Results

### Demographic and clinical characteristics

In this study we reviewed data from 488 patients, including 21 in the OM and 467 in the NOM groups. Clinical characteristics, including sex, clinical stage, pathological type, and treatment methods did not differ significantly between the OM and NOM groups (Student’s *t*-test and Chi-square test). The OM group was significantly older (57.7 ± 12.2 years) than the NOM group (51.5 ± 13.8 years) (*p* < 0.05). There was also a significant difference in history of HBV between the two groups (*p* < 0.05), confirming that ocular metastasis of PLC is associated with history of HBV. The clinical details of all subjects are presented in Table 1.

**TABLE 1:** The clinical characteristics of patients with metastases of primary liver cancer.

Clinical characteristics	OM group (%) (*n* = 21)	NOM group (%) (*n* = 467)	P value^c^
Sex^a^			
Male	16 (76.2)	401 (85.9)	0.219
Female	5 (23.8)	66 (14.1)	
Age (years)^b^			
Mean ± SD	57.7 ± 12.2	51.5 ± 13.8	0.044
History of HBV^a^			
With (+)	18 (85.7)	267 (57.2)	0.018
Without (-)	3 (14.3)	200 (42.8)	
Tumor clinical stage^a^			
Stage 2	0 (0)	15 (3.2)	0.084
Stage 3	3 (14.3)	98 (21.0)	
Stage 4	5 (23.8)	33 (7.1)	
Unknown	13 (61.9)	321 (68.7)	
Pathological type^a^			
Hepatocellular carcinoma (HCC)	0 (0)	38 (8.1)	0.327
Cholangiocarcinoma	0 (0)	24 (5.1)	
Mixed hepatocellular carcinoma	0 (0)	2 (0.4)	
Unknown	21 (0)	403 (86.4)	
Treatment^a^			
Surgery	4 (19.0)	85 (18.2)	0.261
Chemotherapy	3 (14.3)	38 (8.1)	
Protect liver treatment	9 (42.8)	137 (29.3)	
TACE	3 (14.3)	148 (31.6)	
Radiation and chemotherapy	1 (4.8)	7 (1.5)	
Refuse treatment	1 (4.8)	32 (6.8)	
Other	0 (0)	21 (4.5)	

a Chi-square test was used.

b Student’s *t*-test was used.

c Comparison between OM, group and NOM, metastases group.

Notes: *p* < 0.05 was considered statistically significant.

Abbreviations: OM, ocular metastasis; NOM, non-ocular, other sites of metastasis; SD, standard deviation; HBV, hepatitis B virus; TACE, transcatheter arterial chemoembolization.

### Differences in clinical characteristics and risk factors for OM

CEA, CA-199, CA-153, CA-724, TC, TG, HDL, LDL, ApoA1, ApoB, Lp(a), calcium, Hb, ferritin, ALP, and other serological indicators did not differ significantly between the OM and NOM groups, while AFP and CA-125 were significantly higher in the OM group (*p* < 0.001) (Table 2). Binary logistic regression analysis determined that AFP and CA-125 were associated with liver cancer metastasis (Table 3).

**TABLE 2:** The serum indicators of patients with metastases of primary liver cancer.

Serum indicators	OM group	NOM group	*t*	*p* Value
Tumor markers				
AFP (ng/ml)	1,048.80 ± 273.95	559.00 ± 553.48	7.529	<0.001
CEA (ng/ml)	19.78 ± 46.90	19.93 ± 82.97	−0.008	0.994
CA-125 (U/ml)	481.74 ± 356.55	167.26 ± 318.36	4.404	<0.001
CA-199 (U/ml)	221.34 ± 338.41	156.12 ± 292.84	0.992	0.322
CA-153 (u/ml)	18.71 ± 12.76	22.36 ± 25.41	−0.655	0.513
CA-724 (U/ml)	8.60 ± 9.76	6.86 ± 7.16	1.070	0.285
Blood lipid indicators				
TC (mmol/L)	4.63 ± 1.87	4.12 ± 1.46	1.566	0.118
TG (mmol/L)	1.82 ± 1.38	1.24 ± 0.88	1.895	0.072
HDL (mmol/L)	1.57 ± 1.30	1.37 ± 0.95	0.905	0.366
LDL (mmol/L)	2.91 ± 1.97	2.40 ± 1.27	1.148	0.265
ApoA1 (g/L)	1.64 ± 0.47	1.55 ± 0.47	0.825	0.410
ApoB (g/L)	1.01 ± 0.59	0.97 ± 0.58	0.299	0.765
Lp(a) (mg/L)	148.20 ± 182.73	224.15 ± 238.04	−1.407	0.16
Calcium (mmol/L)	2.12 ± 0.24	2.16 ± 0.28	−0.665	0.506
Hb (g/L)	114.71 ± 36.00	116.02 ± 24.02	−0.237	0.812
Ferritin (μg/L)	235.13 ± 243.46	264.73 ± 207.33	−0.635	0.526
ALP (U/L)	225.95 ± 122.46	199.78 ± 189.18	0.628	0.530

Notes: Student’s *t*-test was uesd. *p* < 0.05 represented statistically significant. Abbreviations: OM, ocular metastasis; NOM, non-ocular, other sites of metastasis; TC, total cholesterol; TG, triglycerides; HDL, high-density lipoprotein; LDL, low-density lipoprotein; ApoA1, apolipoprotein A1; ApoB, apolipoprotein B; Lp(a), lipoprotein a; Hb, hemoglobin; ALP, alkaline phosphatase.

**TABLE 3:** Independent risk factors of OM in patients with primary liver cancer.

Factor	B	Exp(B)	OR (95%CI)	*p*
AFP	0.001	1.001	1.001–1.002	<0.001
CA-125	0.001	1.001	1.001–1.002	<0.001

Notes: Binary logistic analysis was used. *p* < 0.05 represented statistically significant. Abbreviations**:** B, coefficient of regression; OR, odds ratio; CI, confidence interval; OM, ocular metastasis.

### Cut-off, sensitivity, specificity, and AUC values of AFP and CA-125 for diagnosing ocular metastases of PLC

**FIGURE 2:**
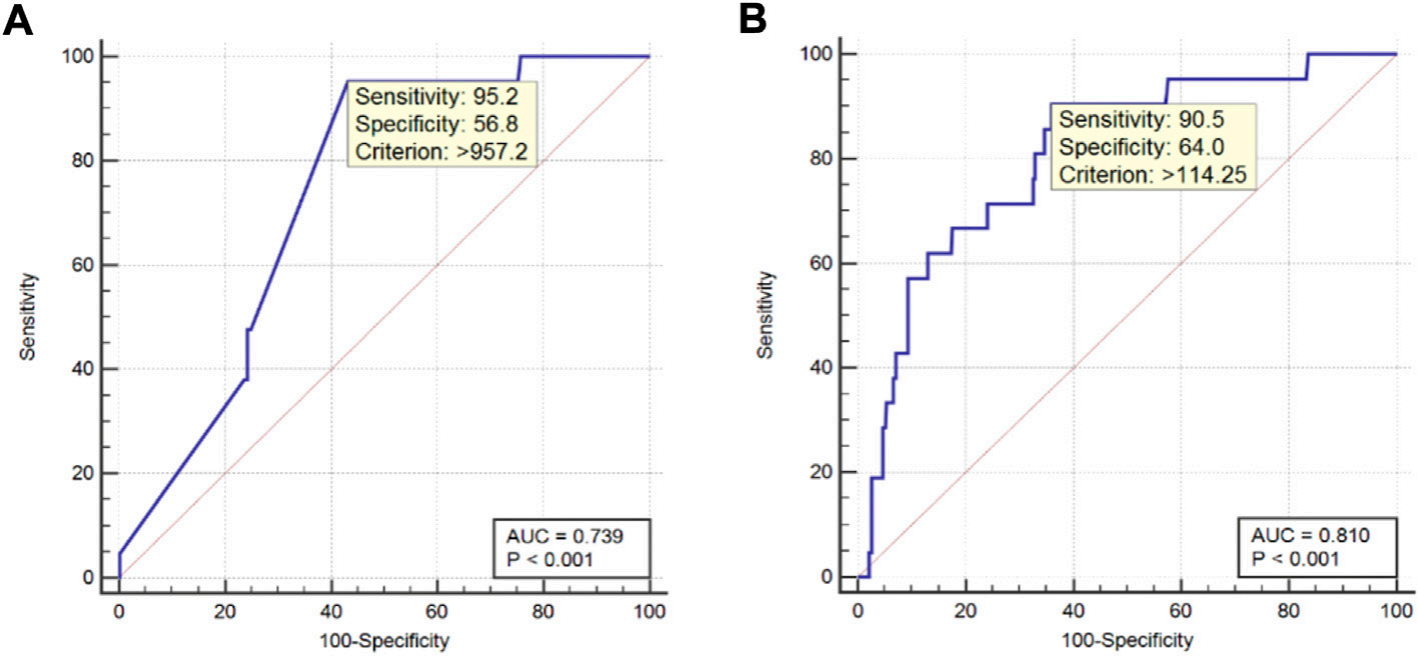
ROC curve of independent risk factors for ocular metastasis from liver cancer. Notes: **(A)**. ROC curve of AFP; **(B)**. ROC curve of CA-125. Abbreviations: AUC, area under the curve; ROC, receiver operating curve.

**FIGURE 3:**
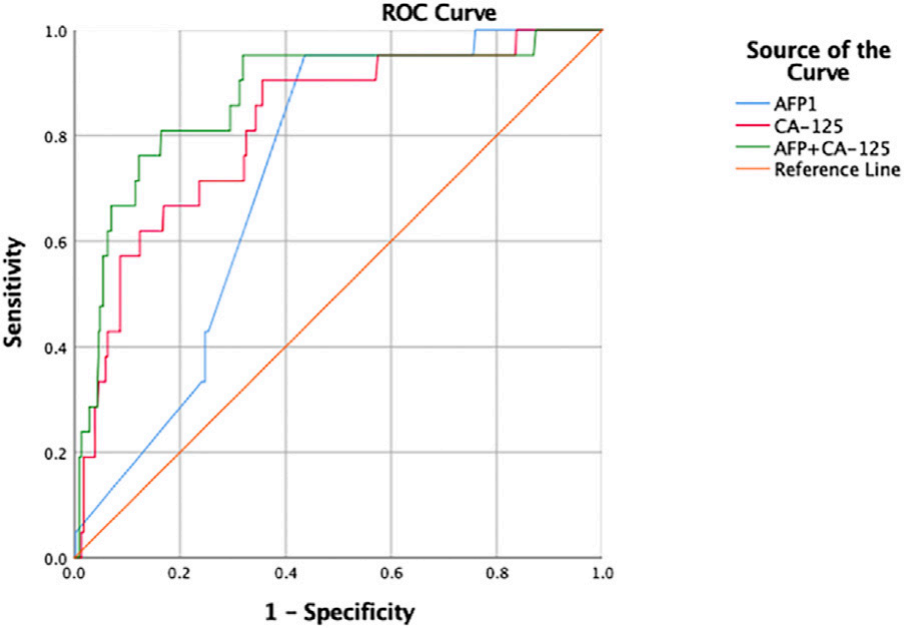
ROC curve of respective and combination of independent risk factors for ocular metastasis from liver cancer. Abbreviations: AUC, area under the curve; ROC, receiver operating curve.

Figure 2 shows the ROC curves for AFP and CA-125, as independent risk factors for ocular metastasis of liver cancer, and their AUC, specificity, sensitivity, and cut-off values. The cut-off values for AFP and CA-125 were 957.2 and 114.25 U/ml and AUC values were 0.739 and 0.810, respectively. AFP had a higher sensitivity value than CA-125, while the specificity of CA-125 was higher than that of AFP. Figure 3 shows a comparison of the ROC curves for AFP, CA-125, and the combination of AFP and CA-125 (AFP + CA-125). The AUC, sensitivity, specificity, and cut-off values for AFP, CA-125, and AFP + CA-125 are presented in Table 4. Among them, AFP + CA-125 had the largest AUC (0.875), AFP the highest sensitivity (95.2%), and AFP + CA-125 the highest specificity (88.4%); all results were statistically significant.

**TABLE 4:** The AUC, sensitivity, specificity and cut-off value for single risk factors in predicting OM from primary liver cancer.

Factor	AUC	Sensitivity (%)	Specificity (%)	Cut-off value U/ml	*p*
AFP	0.739	95.2	56.8	957.2	<0.001
CA-125	0.810	90.2	64.0	14.25	<0.001
AFP + CA-125	0.875	76.2	88.4	—	<0.001

Notes: Sensitivity and specificity were obtained at the point of cut-off value. *p* < 0.05 represented statistically significant. Abbreviations: AUC, area under the curve; OM, ocular metastasis.

## Discussion

Liver carcinoma ranks second among the causes of cancer mortality globally (McGlynn et al., 2015). PLC pathological types include: intrahepatic cholangiocarcinoma (iCCA), HCC, and other rare types (Sia et al., 2017). Among these, HCC is the primary pathological type, accounting for approximately 80% of the total (Adigun et al., 2021). Wang et al. confirmed this through statistical analysis of 2,172 patients with histologically confirmed PLC, among which were 1,823 HCC and 238 iCCA patients, accounting for 83.9 and 11.0%, respectively (Wang et al., 2017). Therefore, we chosed HCC as the main research object.

The incidence and mortality of HCC are increasing. Cirrhosis caused by HBV and hepatitis C is a significant risk factor for HCC globally (Mittal and El-Serag, 2013); however, in most areas of China, HBV is the most important pathogen in the context of PLC. (Li et al., 2015; Ye et al., 2015; Wang et al., 2017). The pathogenesis of HBV is clear. The integrated viral sequence produces different mutations in the preS/S gene, due to replication defects, which can induce various tumorigenesis mechanisms, thereby contributing to HCC (Li et al., 2016).

Infection with HBV can cause a variety of liver lesions and illness; for example, fulminant cirrhosis, chronic hepatitis, hepatic failure, acute hepatitis, and HCC (Liang, 2009). Cirrhosis is an intermediate process, through which viral hepatitis develops into liver cancer. Most HCC patients with a history of viral hepatitis have experienced cirrhosis (Ringelhan et al., 2017). Mahmood et al. (2008) conducted a study on cirrhosis and found that, among the 137 patients with cirrhosis who participated in the trial, the causes of cirrhosis included: hepatitis C virus (61%), HBV (32%), alcoholism (3%), and primary biliary cirrhosis (3%). In addition, 47% of participants had ocular complications. Martín LL et al. (Martín et al., 2021) reported a case of severe multiple and recurrent spontaneous corneal perforation in a patient with primary biliary cirrhosis**.** It is clear that HBV contributes to liver cirrhosis and even liver cancer, and the former can cause ocular complications; therefore, there is evidence for an indirect relationship between history of HBV and ocular lesions. The mechanism underlying this relationship is currently unclear; however, experiments in mice confirmed that SMAD3 mediates the majority of profibrotic activity, since liver cirrhosis, proliferative vitreoretinopathy, and ocular capsule injury are alleviated in SMAD3-null mice (Flanders, 2004).

HBV can also cause eye lesions directly. A 72-year-old chronic HBV patient was also diagnosed with orbital MALT lymphoma, with a clinical manifestation of bilateral ocular protrusion and slight limitation of eye movement, providing evidence for a possible association between HBV and ocular lesions (Lin et al., 2017). There is a risk of eye complications during the prevention and treatment of HBV. HBV vaccine, either alone or administered with other vaccines, appears to be the leading offender in causing uveitis (Benage and Fraunfelder, 2016). Kwon et al. (2001) reported a 15-year-old boy who developed glaucoma while using interferon alpha therapy for chronic HBV. The glaucoma symptoms were improved after discontinuation of interferon therapy.

Liver cancer is frequently occult; 70% of patients with PLC have detectable metastases at initial diagnosis, while metastases account for 90% of the total cancer-related mortality rate (Arvelo et al., 2016). Different distant extrahepatic metastases vary greatly in terms of mortality rates. The lung is common metastatic site of PLC, while brain metastasis is rare, and patients with brain metastasis have the worst prognosis (Wu et al., 2017). Other rarer cases of HCC metastatic sites include thyroid (Liang et al., 2007) and nodal (Liu et al., 2017).

As there is no lymphatic system in the eye, it is rarely a site of malignant tumor metastasis; however, malignant tumors can still be transferred to the eye *via* the blood system. Therefore, blood-rich areas, such as the posterior choroid of the uveal structure, are prone to ocular metastases. The appearance of ocular metastases is also conclusive evidence of tumor metastasis (Konstantinidis and Damato, 2017).

Most patients with stage 4 cancer (distal tumor metastasis), including distant ocular metastases, have clear primary sites (Cohen, 2013). The most common among them for ocular metastases are breast and lung (Konstantinidis and Damato, 2017). Primary cancer sites also include: pancreas (1%), thyroid (1%), prostate (2%), lung carcinoid (2%), cutaneous melanoma (2%), gastrointestinal (GI) tract (4%), kidney (4%), lung (26%), breast (37%), other sites (3%), and unknown (16%) (Shields et al., 2018). All of the patients in our study with eye metastases also had other sites of metastasis. The eye is the terminal organ of liver cancer metastasis. Once metastasis occurs, treatment is difficult and the prognosis poor. Therefore, we compared patients with ocular metastasis to those with other metastasis sites, to identify indicators of ocular metastasis of liver cancer and improve the early diagnosis rate. We found that the OM group was older than the NOM group (*p* < 0.05), likely because elderly patients have a higher prevalence of diabetes, poorer physical fitness, and cannot tolerate higher-intensity treatment (Guo et al., 2017). In addition, due to the association between HBV and ocular lesions, patients with a history of HBV had higher rate of metastases to the eye than to other sites. During clinical diagnosis of PLC, whether the patient has a history of HBV should be determined, to inform subsequent treatment.

Lp(a), ApoB, ApoA1, LDL, HDL, TG, TC, hemoglobin, ferritin, and ALP are commonly used as indicators for evaluating blood lipids and liver function. Statistical analysis did not identify any significant differences between the above indicators in the OM and NOM groups. The serum biomarkers, CA-153, CA-199, CA-125, CEA, AFP, and CA-724 are tumor markers and have clear predictive significance for a variety of tumors (Zou et al., 2006; Zhao et al., 2015; Hogendorf et al., 2017; Kim et al., 2017). In patients with HBV and hepatitis C virus, particularly combined evaluation of AFP, CEA, CA-125, CA-153, and CA-199 have already been implemented (Assmar et al., 2016). Among them, multiple roles for AFP and CA-125 have been confirmed. AFP is a plasma protein produced by embryonic tissue. Healthy adult adults have very low levels of AFP, and some primary cancers can cause significant increases in this factor; therefore, it can be used to screen for tumors and other pathologies in adults (Adigun and Khetarpal, 2019), including primary HCC (Ahmed Mohammed and Roberts, 2017), and gastric cancer (Sun et al., 2017). CA-125 levels are associated with lymphangioleiomyomatosis (LAM), and elevated CA-125 may indicate LAM with pleural effusion, leading to decreased lung function (Glasgow et al., 2018). Karimi-Zarchi et al. (2016) reported that CA-125 can be used for clinical prediction of endometriosis, while another report showed CA-125 as useful for surveillance in ovarian cancer (Esselen et al., 2016).

Non-invasive standard imaging methods, such as MRI, dynamic multi-phase multi-row computed tomography, and ultrasound, are used to diagnose HCC (Wang et al., 2015). Serum biomarker assays are reproducible, simple, and rapid, relative to traditional diagnostic methods. Our statistical analyses confirmed the diagnostic value of AFP and CA-125 for liver metastasis.

Finally, we determined the value of AFP, CA-125, and AFP + CA-125 as diagnostic indicators for ocular metastasis of liver cancer by binary logistic regression analysis and plotting ROC curves. The results indicate that AFP has the highest sensitivity and can be used for early screening of ocular metastases from liver cancer. If AFP rises above 957.2 U/ml, the patient has a higher probability of terminal ocular metastasis. AFP + CA-125 has the highest specificity, and is of great significance for the diagnosis of ocular metastasis from liver cancer.

Our research has certain limitations. First, the sample size of this retrospective study was small, particularly for the OM group. Second, because all patients in the OM group had other sites of metastases simultaneously, confounding factors were inevitable. Furthermore, there were missing items in various clinical statistical analyses, which will have reduced the accuracy of the results. Finally, all subjects were diagnosed and treated in the same hospital, and it is difficult to exclude selection bias; therefore, the accuracy of the conclusions from the results of this study require confirmation in investigations with large samples and multiple centers.

## Conclusion

In summary, we found that patients who had PLC metastasis with a history of HBV were more likely to have ocular metastases than those without. Increases in the serum biomarkers, AFP and CA-125, are associated with an increased likelihood of ocular metastasis. AFP, CA-125, and AFP combined with CA-125 can be used as diagnostic indicators for ocular metastasis in patients with PLC with metastasis.

## Data Availability

The original contributions presented in the study are included in the article/supplementary material, further inquiries can be directed to the corresponding author.
